# Estimating the Oral Absorption from Self-Nanoemulsifying Drug Delivery Systems Using an In Vitro Lipolysis-Permeation Method

**DOI:** 10.3390/pharmaceutics13040489

**Published:** 2021-04-02

**Authors:** Mette Klitgaard, Anette Müllertz, Ragna Berthelsen

**Affiliations:** 1 Department of Pharmacy, University of Copenhagen, 2100 Copenhagen, Denmark; mette.klitgaard@sund.ku.dk; 2 Bioneer: FARMA, Department of Pharmacy, University of Copenhagen, 2100 Copenhagen, Denmark; anette.mullertz@sund.ku.dk

**Keywords:** in vivo–in vitro correlation, lipolysis-permeation, lipid-based drug delivery system, PermeaPad, cinnarizine, lipolysis

## Abstract

The aim of this study was to design an in vitro lipolysis-permeation method to estimate drug absorption following the oral administration of self-nanoemulsifying drug delivery systems (SNEDDSs). The method was evaluated by testing five oral formulations containing cinnarizine (four SNEDDSs and one aqueous suspension) from a previously published pharmacokinetic study in rats. In that study, the pharmacokinetic profiles of the five formulations did not correlate with the drug solubilization profiles obtained during in vitro intestinal lipolysis. Using the designed lipolysis-permeation method, in vitro lipolysis of the five formulations was followed by in vitro drug permeation in Franz diffusion cells equipped with PermeaPad^®^ barriers. A linear in vivo–in vitro correlation was obtained when comparing the area under the in vitro drug permeation–time curve (AUC_0–3h_), to the AUC_0–3h_ of the plasma concentration–time profile obtained from the in vivo study. Based on these results, the evaluated lipolysis-permeation method was found to be a promising tool for estimating the in vivo performance of SNEDDSs, but more studies are needed to evaluate the method further.

## 1. Introduction

The majority of potential drug candidates in the pipelines of the industry today are challenged by their physicochemical properties, such as poor water-solubility, which directly affects the bioavailability of the drug candidates intended for oral administration [1,2,3,4]. Different enabling drug delivery systems have been developed and used to improve the bioavailability of such poorly water-soluble drugs (PWSDs) [2,3,5,6,7,8]. For lipophilic PWSDs, lipid-based drug delivery systems (LbDDSs) represent such enabling drug delivery systems, which have been shown to improve the bioavailability of a range of PWSDs [5,7,8,9,10,11]. LbDDSs, such as the self-nanoemulsifying drug delivery systems (SNEDDSs), consist of different mixtures of lipids, surfactants, and co-solvents. They bypass the dissolution rate-limiting step to oral drug absorption and exploit the endogenous routes of lipid digestion, i.e., lipolysis. 

To aid the development of LbDDSs, multiple in vitro models have been designed to estimate the oral drug performance of LbDDSs. These in vitro models all simulate the human gastro-intestinal (GI) lipolysis process at different levels of complexity [8,12,13]. The most commonly used model is the intestinal in vitro lipolysis model, in which the porcine pancreatic lipase, pancreatin, is typically used as the source of digestive enzymes, because this has been shown to have similar digestive properties to human pancreatic lipase [8,12,13,14,15,16]. The enzyme-induced lipolysis breaks down the lipids and digestible surfactants present in LbDDSs and causes the formation of different colloidal structures that act as a drug solubilization reservoir in equilibrium with the free fraction of solubilized drug. When drugs are administrated in LbDDSs, the utilized excipients commonly need to be digested in order to release the incorporated drug [13,17]. When the intestinal in vitro lipolysis model is used to study the performance of LbDDSs, it is assumed that the amount of drug solubilized in the aqueous phase of the lipolysis medium is available for intestinal absorption, and therefore allows estimation of the drug performance in vivo [8,18,19]. Since its development in 2001 [20], several studies have tried to validate the predictive power of the in vitro lipolysis model. However, as recently described by Feeney et al., only few have succeeded in obtaining an in vivo–in vitro correlation (IVIVC) [8]. Several authors have stressed that the lack of an absorptive step might be an explanation for the general lack of IVIVCs when comparing in vitro lipolysis data with plasma concentration–time profiles, i.e., because the current intestinal in vitro lipolysis model is a closed system, it may not reflect the dynamic interaction between drug solubilization/dissolution, precipitation, and permeation with a continuous absorptive sink present in the human GI tract [3,8,13,21,22,23,24,25,26,27,28,29]. Based on these discussions, designing a method which combines the in vitro lipolysis model with in vitro permeation could possibly improve the estimation of the oral absorption of LbDDSs. There has in fact been an emerging interest in such combined methods, as covered in a review by Berthelsen et al. [12]. In recent studies by Keemink et al. [30,31] and Alskär et al. [32], in vitro lipolysis of LbDDSs was combined with permeation across a Caco-2 monolayer. In this setup, an immobilized microbial lipase was utilized, rather than the more commonly used pancreatin [15,16], because the Caco-2 monolayer was found to be incompatible with the porcine pancreatic extract [30,31,32]. Using their lipolysis-permeation setup, Keemink et al. and Alskär et al. obtained a rank order correlation between the amount of drug permeating the Caco-2 cell monolayer following in vitro lipolysis and the in vivo absorption of orally administered LbDDSs containing the lipophilic drugs fenofibrate and carvedilol [31,32]. 

The majority of drugs have been shown to be absorbed by passive transcellular diffusion [33,34,35]; therefore, an artificial permeation barrier is often considered a sufficiently appropriate alternative barrier for studying drug permeation. In the light of this, Bibi et al. studied the compatibility of an artificial biomimetic barrier, the PermeaPad^®^, which consists of a phospholipid layer sandwiched between two support sheets, in combination with in vitro lipolysis of an SNEDDS in side-by-side diffusion chambers [36]. The integrity of the PermeaPad^®^-barriers was evaluated by the permeation of the hydrophilic permeation marker, calcein, following a lipolysis-permeation study across the same barrier. In that study, it was concluded that the PermeaPad^®^ was compatible with simulated intestinal lipolysis medium, the lipolysis products of the tested SNEDDS, and pancreatin [36].

Another important parameter in the development of lipolysis-permeation methods is the absorption surface area to donor volume (A/V) ratio. In most in vitro permeation models, this is far below that reported for the human intestine, i.e., <0.5 cm^−1^ in vitro vs. 1.9–2.3 cm^−1^ in vivo [35,37,38]. A small A/V ratio might cause an underestimation of drug permeation; therefore, an A/V ratio close to the in vivo A/V ratio might improve the level of in vivo mimicry, as well as the predictability of the in vitro model.

Based on the above, the purpose of the present study was to design and evaluate a simple in vitro lipolysis-permeation method using the PermeaPad^®^ barrier to estimate the oral performance of PWSDs in SNEDDSs. The method was designed using existing equipment, namely, the in vitro lipolysis setup [20,39] and Franz diffusion cells equipped with PermeaPad^®^ barriers. The application of the Franz cells enabled adjusting the donor volume to achieve a high A/V ratio, to simulate the in vivo conditions more closely. Furthermore, sink conditions were secured by applying an acceptor medium with a high drug solubility. 

To evaluate the potential of the designed lipolysis-permeation method, a previously published study by Siqueira et al. was used as a frame of reference [39]. Siqueira et al. performed an in vitro lipolysis study and a pharmacokinetic (PK) study in rats of five oral cinnarizine formulations but were unable to correlate the in vitro and in vivo results [39]. The five studied cinnarizine formulations were four SNEDDSs and one aqueous suspension. In the SNEDDSs, cinnarizine was either dissolved at 80% (*w*/*w*) of its solubility in the preconcentrate (SNEDDS_80%_), suspended at 200% (*w*/*w*) of its solubility in the preconcentrate (superSNEDDS suspension), or dissolved in a supersaturated state corresponding to 200% (*w*/*w*) of its solubility in the preconcentrate (superSNEDDS solution). Additionally, cinnarizine was administrated as an aqueous suspension co-dosed with the blank SNEDDS in a ratio corresponding to administration of the SNEDDS_80%_ (the Chasing principle). While the *in vitro* lipolysis study predicted no difference in the performance of the four SNEDDSs, the PK study showed a different formulation rank-order when comparing the area under the plasma concentration–time curves (AUC), i.e., SNEDDS_80%_ = the Chasing principle > superSNEDDS suspension = superSNEDDS solution = aqueous suspension (Table S1 in the Supplementary Materials) [39]. In the present study, the five formulations from the reference study were tested using the lipolysis-permeation method to evaluate if (i) the in vitro lipolysis results could be reproduced; and if (ii) the method could be used to estimate the oral absorption of cinnarizine from SNEDDSs and thereby obtain an IVIVC for the tested formulations.

## 2. Materials and Methods

### 2.1. Materials

Bovine bile, bovine serum albumin (BSA), calcein, cinnarizine, 4-bromophenylboronic acid (4-BPBA), maleic acid, pancreatin from porcine pancreas (≥3 × USP specifications), propylene glycol, soybean oil, and tris(hydroxymethyl)aminomethane (Tris) were purchased from Sigma Aldrich (St. Louis, MO, USA) at analytical grade. Acetonitrile (ACN), ammonium phosphate monobasic, ethanol absolute, hydrochloric acid (37%), methanol, potassium dihydrogen phosphate, and sodium hydroxide were purchased from VWR Chemicals (Leuven, Belgium). Kolliphor RH 40 and Maisine 35-1 were kindly donated by BASF (Ludwigshafen, Germany) and by Gattefossé (Saint-Priest, France), respectively. Lipoid S PC was obtained from Lipoid (Ludwigshafen, Germany). The PermeaPad^®^ barriers (25 mm) were generously donated by InnoME (Espelkamp, Germany). All water used in the experiments was of purified quality obtained from SG ultra-clear UV apparatus from Holm & Halby Service (Brøndby, Denmark). 

### 2.2. Methods

#### 2.2.1. Media Preparation

The blank simulated intestinal medium (SIM) was prepared by dissolving the components of Table 1 in purified water under stirring at 37 °C overnight. When all components were dissolved, the pH of the medium was adjusted to pH 6.5. 

The hydrophilic marker calcein (logP -1.71, pK_a_ 1.8, 9.2 [40,41]) was dissolved in SIM to reach a concentration of 5 mM (SIM_CAL_) and used to study the integrity of the PermeaPad^®^ barrier in the lipolysis-permeation method. Calcein is an acidic compound; therefore, the pH of SIM_CAL_ was measured after the addition of calcein and re-adjusted to pH 6.5 by the addition of NaOH. SIM_CAL_ was used as the blank donor medium. 

The acceptor medium, PBS_BSA_, was prepared as a 74 mM phosphate buffered saline solution (PBS) (29 mM KH_2_PO_4_ and 45 mM Na_2_HPO_4_⋅7H_2_O) with pH adjusted to 7.4 and supplemented with 4% (*w*/*v*) BSA. The donor and acceptor media were kept iso-osmotic at 290 ± 2 mOsmol/kg to avoid permeation caused by osmosis.

#### 2.2.2. Preparation of Cinnarizine Formulations

To evaluate the designed lipolysis-permeation method, all five formulations from the reference study by Siqueira et al. [39] were tested: the SNEDDS_80%_, the superSNEDDS suspension, the superSNEDDS solution, the Chasing principle, and the aqueous suspension. The blank SNEDDS formulation was prepared from the components listed in Table 2. All formulations were loaded with the PWSD, cinnarizine (logP of 5.03 and pK_a_ of 1.9 and 7.47 [8,39,44,45]).

To prepare the blank SNEDDS, soybean oil, Maisine 35–1, and Kolliphor RH 40 were heated to 50 °C and mixed. After mixing, ethanol was added, and the blank formulation was set to stir at room temperature (25 ± 1 °C) overnight. The drug load was 20 mg/g for the SNEDDS_80%_, and 50 mg/g for the superSNEDDS suspension and superSNEDDS solution. The SNEDDS_80%_ and super-SNEDDS suspensions were prepared by weighing cinnarizine into a glass vial, adding the blank SNEDDS formulation, and stirring the mixture at room temperature overnight. The superSNEDDS solution was prepared by sonicating the cinnarizine with the blank SNEDDS at 60 °C for 2 h followed by storage at 60 °C for 24 h and stirring overnight at 37 °C. The superSNEDDS solution was used within 48 h after preparation to avoid precipitation. To prepare the aqueous suspension (10 mg/mL), cinnarizine was suspended in a 0.5% (*w*/*v*) methylcellulose solution with 5% (*v*/*v*) propylene glycol and set to stir at room temperature (25 ± 1 °C) overnight. For the Chasing principle, blank SNEDDS (with the same lipid load as the SNEDDS_80%_) was added to the lipolysis vessel prior to addition of the aqueous suspension. 

#### 2.2.3. The Lipolysis-Permeation Method

**Figure 1:**
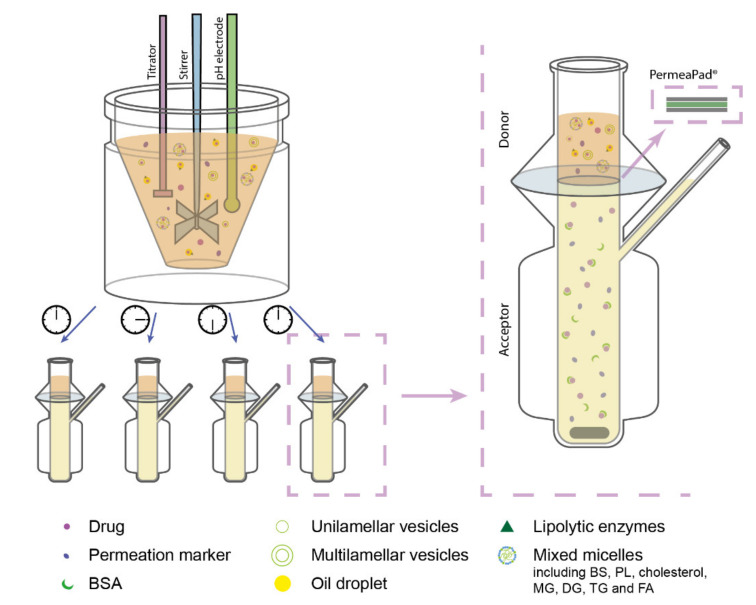
Schematic illustration of the designed lipolysis-permeation method: in vitro lipolysis of an SNEDDS was coupled with permeation across a PermeaPad^®^ barrier in Franz diffusion cells with PBS_BSA_ in the acceptor compartment. Each lipolysis sample contained lipolysis products of the lipid-based drug delivery system (LbDDS) and the permeation marker (calcein) which was embedded in the simulated intestinal medium. Abbreviations: BS, bile salt; BSA, bovine serum albumin; DG, diglycerides; FA, fatty acids; MG, monoglycerides; PBS_BSA_, phosphate-buffered saline (pH 7.4) with 4% (*w*/*v*) BSA; PL, phospholipids; TG, triglycerides.

In the designed lipolysis-permeation method, the established in vitro intestinal lipolysis model was combined with a consecutive drug permeation step across PermeaPad^®^ barriers in Franz diffusion cells (surface area 2 cm^2^, acceptor compartment volume 7 mL) (Figure 1). The Franz diffusion cell acceptor compartment had continuous magnetic stirring and the temperature was kept at 37 °C.

##### Lipolysis Step

The intestinal in vitro lipolysis was carried out as described by Siqueira et al. with one minor modification [39], i.e., a bolus addition of calcium in the SIM (Table 1) was applied instead of the continuous addition of calcium described by Siqueira et al. [39]. In short, the experiments were carried out in a thermostated glass vessel (37 °C), which was set up with pH-stat apparatus (Titrando 804, Metrohm, Herisau, Switzerland) controlling pH input and titration in the Tiamo software v.2.4. Pancreatin from a porcine pancreas was weighed and suspended in SIM (Table 1), mixed, and centrifuged for 8 min at 5500 rpm (44,000× *g* at r_max_) in a Heraeus Megafuge 16R centrifuge from Thermo Fisher Scientific (Osterode, Germany). The supernatant was collected and stored for up to 15 min prior to addition to the lipolysis vessel. The tested formulations were added to the lipolysis vessel to obtain a drug dose of 250 µg/mL cinnarizine and following 15 min of dispersion in 36 mL SIM_CAL_, lipolysis was initiated by the addition of 4 mL of pancreatin, yielding a final lipase activity of 800 USPU/mL. Throughout the in vitro lipolysis, the pH was kept at 6.5 by automatic titration of 0.5 M NaOH. Samples of 2.6 mL were taken at times 0 min (following dispersion, but immediately prior to lipase addition), 15, 30 and 60 min. From each sample, 1 mL was taken out and inhibited with 5 µL lipase inhibitor (1 M 4-BPBA in methanol) to allow for quantification of the drug distribution at the specific time-point, and 1.5 mL of uninhibited sample was transferred to the Franz diffusion cell donor compartment for the permeation step. Each formulation was tested in triplicate (*n* = 3).

##### Sample Treatment

A fraction (50 µL) of each inhibited lipolysis sample was directly diluted in ACN to determine the total amount of drug, i.e., the recovery of added drug. The remaining part of the samples underwent phase separation by centrifugation at 13,300 rpm (170,000× *g* at r_max_) for 15 min in a Thermo Micro CL17 centrifuge from Thermo Fisher Scientific (Osterode, Germany). From each sample, 50 µL of the resulting supernatant (the aqueous phase) was appropriately diluted in ACN. The rest of the aqueous phase was removed, and the pellet was re-suspended in 1 mL ACN and sonicated for 15 min. The re-suspended pellet sample was centrifuged for 15 min at 13,300 rpm and 200 µL of the supernatant appropriately diluted in ACN. The amount of cinnarizine in each diluted sample was quantified by high-performance liquid chromatography (HPLC) (see Section 2.2.4).

##### Permeation Step

For the permeation step, the Franz diffusion cell acceptor compartment was filled with 7 mL of PBS_BSA_. The PermeaPad^®^ barriers (25 mm in diameter) were hydrated by adding 0.5 mL SIM (Table 1) to the donor compartment and allowing the system to equilibrate for 30 min. Following membrane hydration, the SIM was removed and 1.5 mL of uninhibited lipolysis samples (collected after 0, 15, 30, and 60 min of lipolysis, respectively) was added to initiate the permeation study. This way, the permeation from each formulation was tested across a total of twelve PermeaPad^®^ barriers, i.e., drug permeation from each of the four samples collected from each lipolysis replicate was tested. Each permeation study ran for 3 h. Samples of 200 µL were collected from the acceptor compartment at 0, 5, 15, 30, 60, 90, 120, 150, and 180 min. The sample volume was replenished with fresh acceptor medium. Directly following collection, each sample was diluted with equal parts of ACN to precipitate the BSA and centrifuged for 15 min at 13,300 rpm (170,000× *g* at r_max_) in a Thermo Micro CL17 centrifuge from Thermo Fisher Scientific (Osterode, Germany). The supernatants were immediately transferred to HPLC vials for the quantification of cinnarizine, and a 96-well plate for the quantification of calcein by fluorescence detection on a plate reader (see Section 2.2.4 for details).

##### Stability of the PermeaPad^®^ Barrier

A control experiment was conducted to test the stability of the PermeaPad^®^ barrier following prolonged contact with the blank donor medium (SIM_CAL_), and acceptor medium (PBS_BSA_), i.e., without drug formulations and digestive enzymes. The control experiment was conducted following the experimental procedure described in the previous section, using 1.5 mL of SIM_CAL_ as the donor medium. Following 3 h of permeation study, the calcein permeation was quantified as described in Section 2.2.4. 

##### Cinnarizine Solubility

To evaluate if sink conditions were present for cinnarizine using the described lipolysis-permeation method, the apparent solubility of cinnarizine in PBS and PBS_BSA_ was determined by the shake-flask method with an incubation time of 48 h [46].

#### 2.2.4. Quantification Methods

The amount of cinnarizine in the lipolysis-, and permeation samples was quantified by HPLC, using a Dionex Ultimate 3000 pump, an ASI 100 automated sample injector, a p680 pump, a Dionex PDA-100 Photodiode Array Detector and a Dionex Ultimate 3000 Detector from Thermo Scientific (Waltham, MA, USA). All samples were analyzed using a Phenomenex Kinetex C18 column (100 × 4.60 mm, 5 µm) (Torrance, CA, USA). The drug was eluted at 0.8 mL/min with 20 mM ammonium phosphate (pH 4.5):ACN (50:50 (*v*/*v*)). The amounts of cinnarizine in the lipolysis samples were quantified with UV detection at 253 nm. The amounts of cinnarizine in the permeation samples were quantified with fluorescence with excitation and emission wavelengths of 249 and 311 nm, respectively. The lipolysis samples were analyzed using an injection volume of 15 µL and a calibration curve in the range of 50–1000 ng/mL, while permeation samples were analyzed with an injection volume of 50 µL and a calibration curve with the range of 0.5–50 ng/mL. 

The calcein content of the permeation samples was quantified on a Tecan Infinite M200 (Grödig, Austria) plate reader with Tecan Magellan software (ver. 6.5). The samples were analyzed with excitation and emission wavelengths of 485 and 520 nm, respectively, and a gain of 70. The samples were diluted appropriately with a 50:50 (*v*/*v*) mixture of PBS:ACN. The amount of calcein in 200 µL of each diluted sample was quantified with a calibration curve in the range 0.05–4.0 nmol/mL prepared in the same solvent mixture. 

#### 2.2.5. Data Processing

The steady-state flux (*J*) of calcein across the PermeaPad^®^ barriers was determined from the slope of the linear section obtained by plotting the cumulative amount of permeated calcein per surface area of the membrane as a function of time [36,47]. The apparent permeability coefficient (*P_app_*) was calculated from the obtained steady-state flux and the initial concentration of calcein (5 mM) in the donor compartment (*C_0_*), according to Equation (1) [36,47].

(1) $$P_{app}=\frac{J}{C_{0}}$$

In the case of cinnarizine administrated in the different SNEDDSs, the measured concentration in the aqueous phase of the lipolysis medium (i.e., the donor compartment concentration) represents both the free fraction of solubilized drug, and the amount of drug incorporated in the micelles and colloidal structures present in this phase. It is generally assumed that only the free fraction of the drug permeates the intestinal membrane [48]; therefore, the concentration of free drug should be used as *C*_0_ in order to calculate the *P_app_*. However, because it was not possible to quantify the free fraction of cinnarizine in the present setup, the *P_app_* was not calculated for the cinnarizine permeation studies. Rather, the permeation profiles were used for the comparison of the different formulations.

The in vitro AUC_0–3h_ (determined from Figure 4 displaying the mean cumulative permeated amount of cinnarizine as a function of time) was calculated by the linear trapezoidal method.

Statistical analysis of the obtained data was performed using GraphPad Prism ver. 7.04 (GraphPad Software, San Diego, CA, USA). Student’s *t*-test and analysis of variance (ANOVA) were used to compare the means of two or more groups, respectively, with a significance level of α = 0.05. All data are shown as the mean ± standard error of the mean (SEM) for easier comparison with the reference study. The PK parameters from the reference study were determined using WinNonLin ver. 5.2 (Pharsight Corporation, Mountain View, CA, USA). For the present study, the AUC_0–3h_ of the in vivo plasma concentration-time profile was determined from the raw data granted by Siqueria, SD [39] (Table S1) in order to make a direct comparison to the in vitro AUC_0–3h_.

## 3. Results and Discussions

In the present study, a lipolysis-permeation method was designed and evaluated based on: (i) the ability to reproduce the in vitro lipolysis results of the reference study; and (ii) the ability to apply the amount of permeated drug to obtain an IVIVC upon comparison to the in vivo data of the five cinnarizine formulations reported in the reference study by Siqueira et al. [39]. As a control of the permeation barrier integrity, the permeation of calcein was studied throughout the permeation step. Additionally, the A/V ratio and sink conditions were evaluated for the designed method. 

### 3.1. Reproducing In Vitro Lipolysis Results 

**Figure 2:**
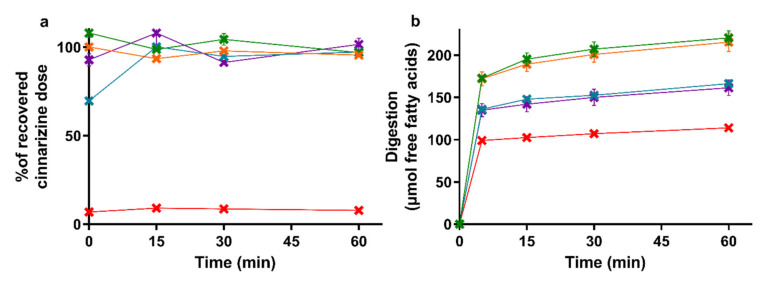
(**a**) Distribution of cinnarizine in the aqueous phase as percent of the total recovered dose after in vitro lipolysis of SNEDDS_80%_ (green), superSNEDDS suspension (blue), superSNEDDS solution (purple), the Chasing principle (orange), and aqueous suspension (red). (**b**) Lipolysis profiles of in vitro lipolysis at pH 6.5 of the same formulations. All data are represented as the mean ± SEM (*n* = 3).

The amount of cinnarizine found in the aqueous phase of the lipolysis samples is depicted in Figure 2a with the accompanying lipolysis profiles of the amount of free fatty acids neutralized with NaOH in Figure 2b. The mean cinnarizine recovery from all lipolysis experiments was 85 ± 3% (mean ± SEM, *n* = 15).

The drug distribution profiles obtained during 60 min of in vitro lipolysis (Figure 2a) are similar to those obtained in the reference study [39]. In both studies, the entire recovered dose was found in the aqueous phase following lipolysis of the four SNEDDS formulations, while the majority of the cinnarizine dose was recovered in the pellet phase for the aqueous suspension. The lipolysis profiles of the titrated amount of free fatty acids released upon digestion of the five formulations (Figure 2b) was rank-ordered according to the amount of lipids added to the lipolysis vessel, i.e., SNEDDS_80%_ = the Chasing principle > superSNEDDS suspension = superSNEDDS solution > aqueous suspension. This is in accordance with the reference study [39], with the only difference being that the continuous calcium addition in the reference study resulted in a higher and more continuous rate of lipolysis. 

### 3.2. Permeation Barrier Integrity

**Figure 3:**
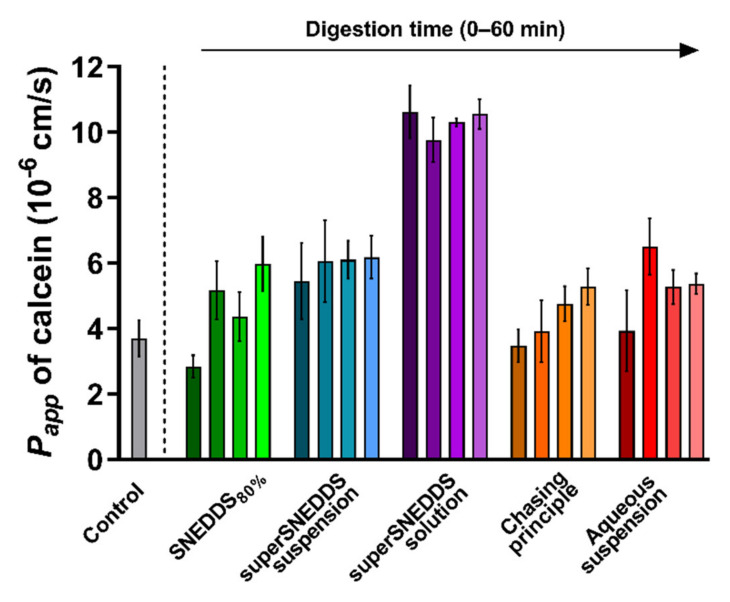
PermeaPad^®^ stability depicted as calcein *P_app_* values calculated from the 3 h of exposure to the different formulations before (0 min lipolysis time, darkest shade of every color) and after digestive enzyme addition (15, 30, and 60 min of lipolysis, the lightest shade of each color represents 60 min of lipolysis). The calcein *P_app_* from the control experiments (grey bar) with no formulation or digestion is depicted for comparison. All data are depicted as the mean ± SEM (*n* = 3).

The integrity of the PermeaPad^®^ barrier was tested following prolonged contact with the blank donor medium containing calcein (SIM_CAL_) and acceptor medium (PBS_BSA_), as well as during exposure to the lipolysis samples for each of the five formulations. Barrier integrity was evaluated based on the observed *P_app_* of calcein depicted in Figure 3. The barrier integrity was tested for each formulation without (0 min lipolysis) and with enzymatic lipolysis (15–60 min lipolysis). The individual calcein permeation profiles can be found in the Supplementary Materials, Figure S1.

In the control experiment with SIM_CAL_ as the donor medium and PBS_BSA_ as the acceptor medium, the *P_app_* of calcein across the PermeaPad^®^ barrier was 3.7 ± 0.6 × 10^−6^ cm/s. This value is not significantly different from the values reported by Bibi et al. (*P_app_* 3.4 ± 0.5 × 10^−6^ cm/s [36]), which indicates that PermeaPad^®^ barrier was compatible with the selected donor and acceptor media. 

When comparing the calcein *P_app_* values obtained in the presence of each digesting formulation with the calcein *P_app_* of the blank control (SIM_CAL_), no difference was observed, except for a significantly higher *P_app_* (*p* ≤ 0.05) in the presence of the superSNEDDS solution (at all four time-points) (Figure 3). Generally, a slight tendency towards an increased *P_app_* of calcein in the presence of the digestive enzymes (0 min lipolysis time compared to 15–60 min of lipolysis) was observed for all formulations, although this difference was not found to be significant (Figure 3, Figure S1). 

The calcein *P_app_* in the presence of the superSNEDDS solution was significantly higher when compared the calcein *P_app_* from the present control study, which might indicate that the permeation barrier in these studies was disrupted to some degree. However, because all values were lower than the calcein *P_app_* of 1.65 ± 0.1 × 10^−5^ cm/s previously reported for the PermeaPad^®^ barrier with no lipid barrier layer [49], it was concluded that some barrier function was retained throughout the study. This was additionally based on the observation that the higher calcein *P_app_* did not affect the permeation of cinnarizine (Section 3.3). Based on these results, it was concluded that permeation barrier function was sufficiently retained during all conducted studies.

### 3.3. Permeation Profiles

**Figure 4:**
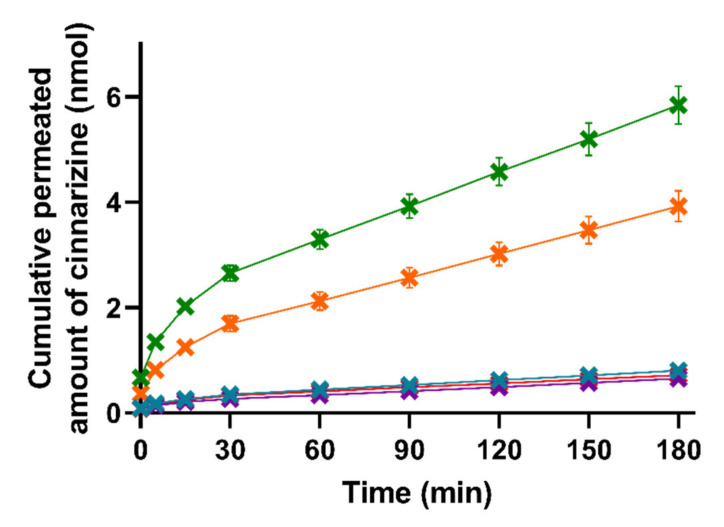
Mean cumulative permeated amount of cinnarizine as a function of time when studying the five formulations SNEDDS_80%_ (green), superSNEDDS suspension (blue), superSNEDDS solution (purple), the Chasing principle (orange), and aqueous suspension (red) in the lipolysis-permeation method. The data are presented as the mean ± SEM (*n* = 12).

The permeation of cinnarizine across the PermeaPad^®^ barrier was determined for lipolysis samples taken at each of the four time-points (0, 15, 30, and 60 min of lipolysis) for all five formulations. The individual cinnarizine permeation profiles for each formulation after each lipolysis time-point can be found in Figure S2. As can be seen in Figure S2, lipolysis had no significant effect on the permeation of cinnarizine and, therefore, the permeation profiles depicted in Figure 4 represent a pooled mean ± SEM for each formulation (*n* = 12). 

The lipase-induced lipolysis seemingly did not affect the amount of cinnarizine permeation in the present study; therefore, the need for the lipolysis step could be challenged and perhaps rather substituted with a dispersion of the formulations in SIM, as has been used in other studies [22]. This is in accordance with studies by Michaelsen et al., who showed that addition of the lipase inhibitor Orlistat to SNEDDSs did not change the AUC of the plasma concentration–time profile of halofantrine and fenofibrate after oral dosing to rats [50,51]. However, other LbDDSs or drugs might be affected differently by lipid digestion. 

As can be seen in Figure 4, there were no significant differences in the amount of permeated cinnarizine from the lipolysis samples of the superSNEDDS suspension, superSNEDDS solution, and the aqueous suspension at any given time point (*t* = 5–180 min). These results indicate that cinnarizine very likely precipitated in the donor compartment following administration of the superSNEDDS suspension and superSNEDDS solution. Following precipitation, the amount of cinnarizine in solution was expected to be close to that obtained following administration of the aqueous suspension, because the amount of permeated drug for these three formulations was comparable (i.e., the superSNEDDS suspension, superSNEDDS solution, and the aqueous suspension, Figure 4), and the concentration gradient across the permeation barrier, therefore, must be assumed to be similar. In the case of the SNEDDS_80%_ and the Chasing principle, a significantly higher amount of cinnarizine (*p* ≤ 0.05) permeated from these formulations throughout the experiment (*t* = 5–180 min) when compared to the other three formulations (Figure 4). This observed difference is expected to be caused by the presence of a high amount of lipids and surfactants in these formulations, which inhibited the precipitation of cinnarizine following dispersion and digestion in SIM. The amount of permeated cinnarizine was higher from the SNEDDS_80%_ and the Chasing principle; thus, it is suggested that the distribution between the amount of drug solubilized in the SNEDDS and/or the colloidal structures formed during digestion of the SNEDDS and the amount of drug in aqueous solution (i.e., the free fraction) equilibrate much faster following drug absorption, as compared to the distribution between precipitated/undissolved cinnarizine and the amount of cinnarizine in aqueous solution (i.e., in the case if the superSNEDDS suspension, superSNEDDS solution, and the aqueous suspension).

Considering the PK parameters from the in vivo study (Table S1), only the AUC_0–3h_ was significantly different between the formulations, making this parameter most relevant for the comparison with the in vitro lipolysis-permeation results. The rank-order of the formulations based on the AUC_0–3h_ of the permeation profiles obtained from the in vitro lipolysis-permeation experiments is comparable to the rank order of the in vivo AUC_0–3h_ (Figure 4, Table S1), i.e., SNEDDS_80%_ > the Chasing principle > superSNEDDS suspension = superSNEDDS solution = aqueous suspension. The SNEDDS_80%_ did, however, result in a significantly higher amount of in vitro permeation (higher in vitro AUC_0–3h_) compared to the Chasing principle (*p* ≤ 0.05), while there was no difference in the in vivo AUC_0–3h_ values of these two formulations (Figure 4, Table S1). The observed lower drug permeation from the Chasing principle might be due to a lower dissolution/solubilization rate in vitro compared to in vivo. Specifically, this difference might be caused by the lack of a gastric step in the applied lipolysis-permeation method. A gastric step could increase the amount of drug available for permeation by increasing the amount of solubilized drug due to higher cinnarizine solubility at gastric pH, longer incubation time, and pre-lipolysis by gastric lipase. In a recent study by Klitgaard et al., the benefit of simulating the gastric lipolysis in combination with the intestinal lipolysis was shown [52]; however, the specific effect of this additional step in relation to the designed lipolysis-permeation method is for future studies to conclude. 

### 3.4. In Vivo–In Vitro Correlation

**Figure 5:**
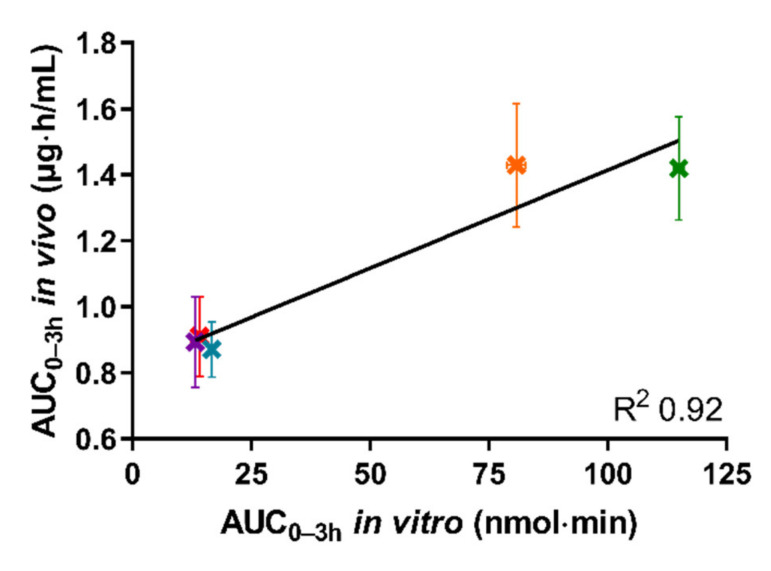
The correlation between in vitro AUC_0–3h_ of permeated cinnarizine in the lipolysis-permeation method and in vivo AUC_0-3h_ from the reference study of the SNEDDS_80%_ (green), superSNEDDS suspension (blue), superSNEDDS solution (purple), the Chasing principle (orange), and aqueous suspension (red). The in vitro data are presented as the mean ± SEM (*n* = 12), and the in vivo data as mean ± SEM (*n* = 6). Due to low variability of the in vitro data, these deviations are barely visible.

Figure 5 depicts the in vivo AUC_0–3h_ of the plasma concentration–time curve obtained following oral administration of the five tested formulations in rats, as a function of the in vitro AUC_0–3h_ obtained from the permeated amount of drug from the same five formulations in the lipolysis-permeation method in this study.

As can be seen in Figure 5, a coefficient of determination (*R*^2^) of 0.92 was obtained by linear correlation. The reason that the *R*^2^ is not higher is the lower in vitro AUC_0–3h_ observed for the Chasing principle compared to the SNEDDS_80%_, as described above. However, the amount of drug permeated using the lipolysis-permeation method (in vitro AUC_0-3h_) displayed a good correlation with the in vivo AUC_0–3h_. In the donor compartment, there will be an equilibrium between cinnarizine in the formed colloidal structures, e.g., vesicles and mixed micelles [53,54], and the free fraction available for absorption. The presence of the permeation barrier enables mass transfer of the free fraction, thereby enabling the dynamic interaction between the free fraction, solubilized fraction, and the permeated drug for which multiple studies have indicated a need [3,8,13,21,22,23,24,25,26,27,28,29,55,56]. 

### 3.5. Absorption Surface Area to Donor Volume Ratio and Sink Conditions of the Lipolysis-Permeation Method

During the initial design of the lipolysis-permeation method, special focus was on (i) combining lipolysis and permeation in a way that ensured a high A/V ratio; and (ii) ensuring sink conditions in the acceptor compartment of the permeation module. Designing the lipolysis-permeation method using Franz diffusion cells with a vertical permeation setup prompted the possibility to use a low donor volume. It was thereby possible to obtain an A/V ratio of 1.34 cm^−1^, which more closely simulated the in vivo A/V ratio (1.9–2.3 cm^−1^) than previous permeation studies with A/V ratios of <0.5 cm^−1^ [35,37,38]. The addition of 4% (*w*/*v*) BSA to the acceptor medium (PBS) significantly improved the apparent solubility of cinnarizine (*p* ≤ 0.05) with 1.30 ± 0.02 µg/mL in PBS_BSA_, compared to 0.07 ± 0.02 µg/mL in PBS. With this increase in apparent drug solubility, sink conditions were ensured for the present experimental setup. This was confirmed because the highest amount of drug permeating resulted in a concentration of 13% (*w*/*w*) of the apparent saturation solubility in the acceptor medium PBS_BSA_. In combination, the high A/V ratio and ensured sink conditions resulted in a permeation of 0.08–0.75% of the cinnarizine added to the donor compartment (Figure 4). This is a clear increase when compared to the similar setup used by Bibi et al., which resulted in a 0.00012% permeation from the dosed cinnarizine SNEDDS [36]. However, more studies are needed to evaluate the effect of a higher A/V ratio and improved sink conditions.

## 4. Conclusions

In the present study, an in vitro lipolysis-permeation method to estimate the oral drug absorption following administration of an SNEDDS was designed. The lipolysis-permeation method had an A/V ratio close to the in vivo conditions and enabled the use of physiologically relevant SIM and enzymes by applying the PermeaPad^®^ barrier. Furthermore, sink conditions were ensured by the addition of 4% (*w*/*v*) BSA in the acceptor compartment. The predictability of the lipolysis-permeation method was evaluated using PK data from a reference study, in which five cinnarizine formulations were tested in rats [39]. No correlation was obtained between the AUC_0–60min_ of the drug solubilization profiles during in vitro lipolysis and the in vivo PK data, which is in accordance with the reference study. However, the in vitro AUC_0–3h_ of the permeation profiles from the five formulations showed a linear rank order correlation with the in vivo AUC_0–3h_ of the plasma concentration time profiles. Based on this, the designed in vitro lipolysis-permeation method was found to be a promising tool for predicting the oral absorption of SNEDDSs, but further studies are needed to truly evaluate the method.

## Figures and Tables

**Table 1:** Composition of simulated intestinal in vitro lipolysis medium (SIM) [39,42,43].

Component	Concentration (mM)
Bovine Bile	2.95
Phospholipids	0.26
NaCl	146.8
Tris	2
Maleic acid	2
CaCl_2_·2H_2_O	1.4

**Table 2:** Composition of the blank self-nanoemulsifying drug delivery system (SNEDDS) formulation.

Component	Ratio (% *w*/*w*)
Soybean oil: Maisine 35-1 (1:1, weight ratio)	55
Kolliphor RH 40	35
Ethanol	10

## Data Availability

Data is contained within the article or Supplementary Materials.

## Supplementary Materials

The following are available online at https://www.mdpi.com/article/10.3390/pharmaceutics13040489/s1, Table S1: Pharmacokinetic parameters for cinnarizine after the administration of 25 mg/kg cinnarizine to rats, adapted from the reference study. Figure S1: The individual permeation graphs with the cumulative permeated amount of calcein after testing the five formulations in the lipolysis-permeation method. Figure S2: The individual permeation graphs with the cumulative permeated amount of cinnarizine after testing the five formulations in the lipolysis-permeation method. Reference [39] is cited in the Supplementary Materials.

## References

[1] Ruiz-Garcia A., Bermejo M., Moss A., Casabo V.G. (2008). Pharmacokinetics in drug discovery. J. Pharm. Sci..

[2] Fagerberg J.H., Bergström C.A.S. (2015). Intestinal solubility and absorption of poorly water soluble compounds: Predictions, challenges and solutions. Ther. Deliv..

[3] Kostewicz E.S., Abrahamsson B., Brewster M., Brouwers J., Butler J., Carlert S., Dickinson P.A., Dressman J., Holm R., Klein S. (2014). In vitro models for the prediction of in vivo performance of oral dosage forms. Eur. J. Pharm. Sci..

[4] Zaki N.M., Artursson P., Bergstroöm C.A.S. (2010). A Modified physiological BCS for prediction of intestinal absorption in drug discovery. Mol. Pharm..

[5] Porter C.J., Charman W.N. (2001). In vitro assessment of oral lipid based formulations. Adv. Drug Deliv. Rev..

[6] Carrière F. (2016). Impact of gastrointestinal lipolysis on oral lipid-based formulations and bioavailability of lipophilic drugs. Biochimie.

[7] Porter C.J., Pouton C.W., Cuine J.F., Charman W.N. (2008). Enhancing intestinal drug solubilisation using lipid-based delivery systems. Adv. Drug Deliv. Rev..

[8] Feeney O.M., Crum M.F., McEvoy C.L., Trevaskis N.L., Williams H.D., Pouton C.W., Charman W.N., Bergström C.A., Porter C.J. (2016). 50 years of oral lipid-based formulations: Provenance, progress and future perspectives. Adv. Drug Deliv. Rev..

[9] Christophersen P.C., Christiansen M.L., Holm R., Kristensen J., Jacobsen J., Abrahamsson B., Müllertz A. (2014). Fed and fasted state gastro-intestinal in vitro lipolysis: In vitro in vivo relations of a conventional tablet, a SNEDDS and a solidified SNEDDS. Eur. J. Pharm. Sci..

[10] Dahan A., Hoffman A. (2007). The effect of different lipid based formulations on the oral absorption of lipophilic drugs: The ability of in vitro lipolysis and consecutive ex vivo intestinal permeability data to predict in vivo bioavailability in rats. Arbeitsgemeinschaft fur Pharmazeutische Verfahrenstechnik/Eur. J. Pharm. Biopharm..

[11] Larsen A.T., Ohlsson A.G., Polentarutti B., Barker R.A., Phillips A.R., Abu-Rmaileh R., Dickinson P.A., Abrahamsson B., Østergaard J., Müllertz A. (2013). Oral bioavailability of cinnarizine in dogs: Relation to SNEDDS droplet size, drug solubility and in vitro precipitation. Eur. J. Pharm. Sci..

[12] Berthelsen R., Klitgaard M., Rades T., Müllertz A. (2019). In vitro digestion models to evaluate lipid based drug delivery systems; present status and current trends. Adv. Drug Deliv. Rev..

[13] Berthelsen R., Sassene P., Rades T., Müllertz A., Müllertz A., Perrie Y., Rades T. (2016). Evaluating oral drug delivery systems: Digestion models. Analytical Techniques in the Pharmaceutical Sciences.

[14] Williams H.D., Sassene P., Kleberg K., Bakala-N’Goma J.-C., Calderone M., Jannin V., Igonin A., Partheil A., Marchaud D., Jule E. (2012). Toward the establishment of standardized in vitro tests for lipid-based formulations, part 1: Method parameterization and comparison of in vitro digestion profiles across a range of representative formulations. J. Pharm. Sci..

[15] Verger R., Borgström B., Brockman H.L. (1984). Pancreatic lipases. Lipases.

[16] Capolino P., Guérin C., Paume J., Giallo J., Ballester J.-M., Cavalier J.-F., Carrière F. (2011). In vitro gastrointestinal lipolysis: Replacement of human digestive lipases by a combination of rabbit gastric and porcine pancreatic extracts. Food Dig..

[17] Palin K.J., Wilson C.G. (1984). The effect of different oils on the absorption of probucol in the rat. J. Pharm. Pharmacol..

[18] Larsen A., Holm R., Pedersen M.L., Müllertz A. (2008). Lipid-based formulations for danazol containing a digestible surfactant, labrafil M2125CS: In vivo bioavailability and dynamic in vitro lipolysis. Pharm. Res..

[19] Dahan A., Hoffman A. (2006). Use of a dynamic in vitro lipolysis model to rationalize oral formulation development for poor water soluble drugs: Correlation with in vivo data and the relationship to intra-enterocyte processes in rats. Pharm. Res..

[20] Zangenberg N.H., Mullertz A., Kristensen H.G., Hovgaard L. (2001). A dynamic in vitro lipolysis model. I. Controlling the rate of lipolysis by continuous addition of calcium. Eur. J. Pharm. Sci..

[21] Lee K.W.Y., Porter C.J.H., Boyd B.J. (2013). The effect of administered dose of lipid-based formulations on the in vitro and in vivo performance of cinnarizine as a model poorly water-soluble drug. J. Pharm. Sci..

[22] Griffin B.T., Kuentz M., Vertzoni M., Kostewicz E.S., Fei Y., Faisal W., Stillhart C., O’Driscoll C.M., Reppas C., Dressman J.B. (2014). Comparison of in vitro tests at various levels of complexity for the prediction of in vivo performance of lipid-based formulations: Case studies with fenofibrate. Arbeitsgemeinschaft fur Pharmazeutische Verfahrenstechnik/Eur. J. Pharm. Biopharm..

[23] Stillhart C., Kuentz M. (2016). Trends in the assessment of drug supersaturation and precipitation in vitro using lipid-based delivery systems. J. Pharm. Sci..

[24] Alskär L.C., Bergström C.A.S. (2015). Models for predicting drug absorption from oral lipid-based formulations. Curr. Mol. Biol. Rep..

[25] Stillhart C., Imanidis G., Kuentz M. (2013). Insights into drug precipitation kinetics during in vitro digestion of a lipid-based drug delivery system using in-line raman spectroscopy and mathematical modeling. Pharm. Res..

[26] Bevernage J., Brouwers J., Annaert P., Augustijns P. (2012). Drug precipitation–permeation interplay: Supersaturation in an absorptive environment. Arbeitsgemeinschaft fur Pharmazeutische Verfahrenstechnik/Eur. J. Pharm. Biopharm..

[27] Thomas N., Holm R., Müllertz A., Rades T. (2012). In vitro and in vivo performance of novel supersaturated self-nanoemulsifying drug delivery systems (super-SNEDDS). J. Control. Release.

[28] Buckley S.T., Fischer S.M., Fricker G., Brandl M. (2012). In vitro models to evaluate the permeability of poorly soluble drug entities: Challenges and perspectives. Eur. J. Pharm. Sci..

[29] Hens B., Brouwers J., Corsetti M., Augustijns P. (2015). Gastrointestinal behavior of nano- and microsized fenofibrate: In vivo evaluation in man and in vitro simulation by assessment of the permeation potential. Eur. J. Pharm. Sci..

[30] Keemink J., Bergström C.A.S. (2018). Caco-2 cell conditions enabling studies of drug absorption from digestible lipid-based formulations. Pharm. Res..

[31] Keemink J., Mårtensson E., Bergström C.A.S. (2019). Lipolysis-permeation setup for simultaneous study of digestion and ab-sorption in vitro. Mol. Pharm..

[32] Alskär L.C., Parrow A., Keemink J., Johansson P., Abrahamsson B., Bergström C.A. (2019). Effect of lipids on absorption of carvedilol in dogs: Is coadministration of lipids as efficient as a lipid-based formulation?. J. Control. Release.

[33] Mandagere A.K., Thompson T.N., Hwang K.-K. (2002). Graphical model for estimating oral bioavailability of drugs in humans and other species from their caco-2 permeability and in vitro liver enzyme metabolic stability rates. J. Med. Chem..

[34] Florence A.T. (2011). Physicochemical Principles of Pharmacy.

[35] Berben P., Bauer-Brandl A., Brandl M., Faller B., Flaten G.E., Jacobsen A.-C., Brouwers J., Augustijns P. (2018). Drug permeability profiling using cell-free permeation tools: Overview and applications. Eur. J. Pharm. Sci..

[36] Bibi H.A., Holm R., Bauer-Brandl A. (2017). Simultaneous lipolysis/permeation in vitro model, for the estimation of bioavailability of lipid based drug delivery systems. Arbeitsgemeinschaft fur Pharmazeutische Verfahrenstechnik/Eur. J. Pharm. Biopharm..

[37] Sironi D., Christensen M., Rosenberg J., Bauer-Brandl A., Brandl M. (2017). Evaluation of a dynamic dissolution/permeation model: Mutual influence of dissolution and barrier-flux under non-steady state conditions. Int. J. Pharm..

[38] Mudie D.M., Shi Y., Ping H., Gao P., Amidon G.L., Amidon G.E. (2012). Mechanistic analysis of solute transport in anin vitrophysiological two-phase dissolution apparatus. Biopharm. Drug Dispos..

[39] Siqueira S.D., Müllertz A., Gräeser K., Kasten G., Mu H., Rades T. (2017). Influence of drug load and physical form of cinnarizine in new SNEDDS dosing regimens: In vivo and in vitro evaluations. AAPS J..

[40] Naderkhani E., Isaksson J., Ryzhakov A., Flaten G.E. (2014). Development of a biomimetic phospholipid vesicle-based permeation assay for the estimation of intestinal drug permeability. J. Pharm. Sci..

[41] Flaten G.E., Dhanikula A.B., Luthman K., Brandl M. (2006). Drug permeability across a phospholipid vesicle based barrier: A novel approach for studying passive diffusion. Eur. J. Pharm. Sci..

[42] Sassene P., Kleberg K., Williams H.D., Bakala-N’Goma J.-C., Carriere F., Calderone M., Jannin V., Igonin A., Partheil A., Marchaud D. (2014). Toward the establishment of standardized in vitro tests for lipid-based formulations, part 6: Effects of varying pancreatin and calcium levels. AAPS J..

[43] Mosgaard M.D., Sassene P., Mu H., Rades T., Müllertz A. (2015). Development of a high-throughput in vitro intestinal lipolysis model for rapid screening of lipid-based drug delivery systems. Arbeitsgemeinschaft fur Pharmazeutische Verfahrenstechnik/Eur. J. Pharm. Biopharm..

[44] Khan J., Rades T., Boyd B.J. (2016). Lipid-based formulations can enable the model poorly water-soluble weakly basic drug cinnarizine to precipitate in an amorphous-salt form during in vitro digestion. Mol. Pharm..

[45] Gu C., Rao D., Gandhi R.B., Hilden J., Raghavan K. (2005). Using a novel multicompartment dissolution system to predict the effect of gastric pH on the oral absorption of weak bases with poor intrinsic solubility. J. Pharm. Sci..

[46] Berthelsen R., Sjögren E., Jacobsen J., Kristensen J., Holm R., Abrahamsson B., Müllertz A. (2014). Combining in vitro and in silico methods for better prediction of surfactant effects on the absorption of poorly water soluble drugs—A fenofibrate case example. Int. J. Pharm..

[47] Di Cagno M., Bibi H.A., Bauer-Brandl A. (2015). New biomimetic barrier Permeapad™ for efficient investigation of passive permeability of drugs. Eur. J. Pharm. Sci..

[48] Dahan A., Miller J.M. (2012). The solubility–Permeability interplay and its implications in formulation design and development for poorly soluble drugs. AAPS J..

[49] Bibi H.A., Di Cagno M., Holm R., Bauer-Brandl A. (2015). Permeapad™ for investigation of passive drug permeability: The effect of surfactants, co-solvents and simulated intestinal fluids (FaSSIF and FeSSIF). Int. J. Pharm..

[50] Michaelsen M.H., Wasan K.M., Sivak O., Müllertz A., Rades T. (2015). The effect of digestion and drug load on halofantrine absorption from self-nanoemulsifying drug delivery system (SNEDDS). AAPS J..

[51] Michaelsen M.H., Jørgensen S.D.S., Abdi I.M., Wasan K.M., Rades T., Müllertz A. (2019). Fenofibrate oral absorption from SNEDDS and super-SNEDDS is not significantly affected by lipase inhibition in rats. Eur. J. Pharm. Biopharm..

[52] Klitgaard M., Beilles S., Sassene P.J., Berthelsen R., Müllertz A. (2020). Adding a gastric step to the intestinal in vitro digestion model improves the prediction of pharmacokinetic data in beagle dogs of two lipid-based drug delivery systems. Mol. Pharm..

[53] Tran T., Siqueira S.D., Amenitsch H., Rades T., Müllertz A. (2017). Monoacyl phosphatidylcholine inhibits the formation of lipid multilamellar structures during in vitro lipolysis of self-emulsifying drug delivery systems. Eur. J. Pharm. Sci..

[54] Tran T., Fatouros D.G., Vertzoni M., Reppas C., Müllertz A. (2017). Mapping the intermediate digestion phases of human healthy intestinal contents from distal ileum and caecum at fasted and fed state conditions. J. Pharm. Pharmacol..

[55] Li S., He H., Parthiban L.J., Yin H., Serajuddin A.T. (2005). IV-IVC considerations in the development of immediate-release oral dosage form. J. Pharm. Sci..

[56] Buch P., Langguth P., Kataoka M., Yamashita S. (2009). IVIVC in oral absorption for fenofibrate immediate release tablets using a dissolution/permeation system. J. Pharm. Sci..

