# Supplementary material for: Estimating the Oral Absorption from Self-Nanoemulsifying Drug Delivery Systems Using an In Vitro Lipolysis-Permeation Method
Source: Pharmaceutics. 2021 Apr 2;13(4):489. doi: 10.3390/pharmaceutics13040489 (PMC8065752; doi:10.3390/pharmaceutics13040489)
Supplement: Supplementary file 1 [file pharmaceutics-13-00489-s001.pdf]

pharmaceutics

MDPI

Supplementary Materials

Estimating the Oral Absorption from Self-Nanoemulsifying Drug Delivery Systems Using an In Vitro Lipolysis-Permeation Method

Mette Klitgaard, Anette Müllertz and Ragna Berthelsen

Table S1. Pharmacokinetic parameters for cinnarizine after administration of 25 mg/kg cinnarizine to rats. The table has been adapted from the reference study by Siqueria et al. [39], and $AUC_{0–3h}$ added to the table with permission from Springer Nature.

Pharmacokinetic Parameter	$SNEDDS_{80\%}$	SuperSNEDDS Suspension	SuperSNEDDS Solution	Chasing Principle	Aqueous Suspension
C_{max} (µg/mL)	0.8 ± 0.1	0.5 ± 0.0	0.5 ± 0.1	0.9 ± 0.1	0.5 ± 0.1
t_{max} (h)	1.5 ± 0.1	2.1 ± 0.4	1.7 ± 0.2	1.5 ± 0.0	1.8 ± 0.3
$AUC_{0–6h}$ (µg·h/mL)	2.4 ±0.2 ^{a}	1.7 ± 0.3	1.7 ± 0.1	2.4 ± 0.2 ^{a}	1.4 ± 0.2 ^{b}
$AUC_{0–3h}$ (µg·h/mL)	1.4 ± 0.2^{a}	0.9 ± 0.1^{b}	0.9 ± 0.1^{b}	1.4 ± 0.2^{a}	0.9 ± 0.1^{b}

Data are reported as mean ± SEM (n = 6). ^{a} indicates a statistically significant difference to ^{b} ($p < 0.05$).

Publisher's Note: MDPI stays neutral with regard to jurisdictional claims in published maps and institutional affiliations.

Copyright: © 2020 by the authors. Submitted for possible open access publication under the terms and conditions of the Creative Commons Attribution (CC BY) license (http://creativecommons.org/licenses/by/4.0/).

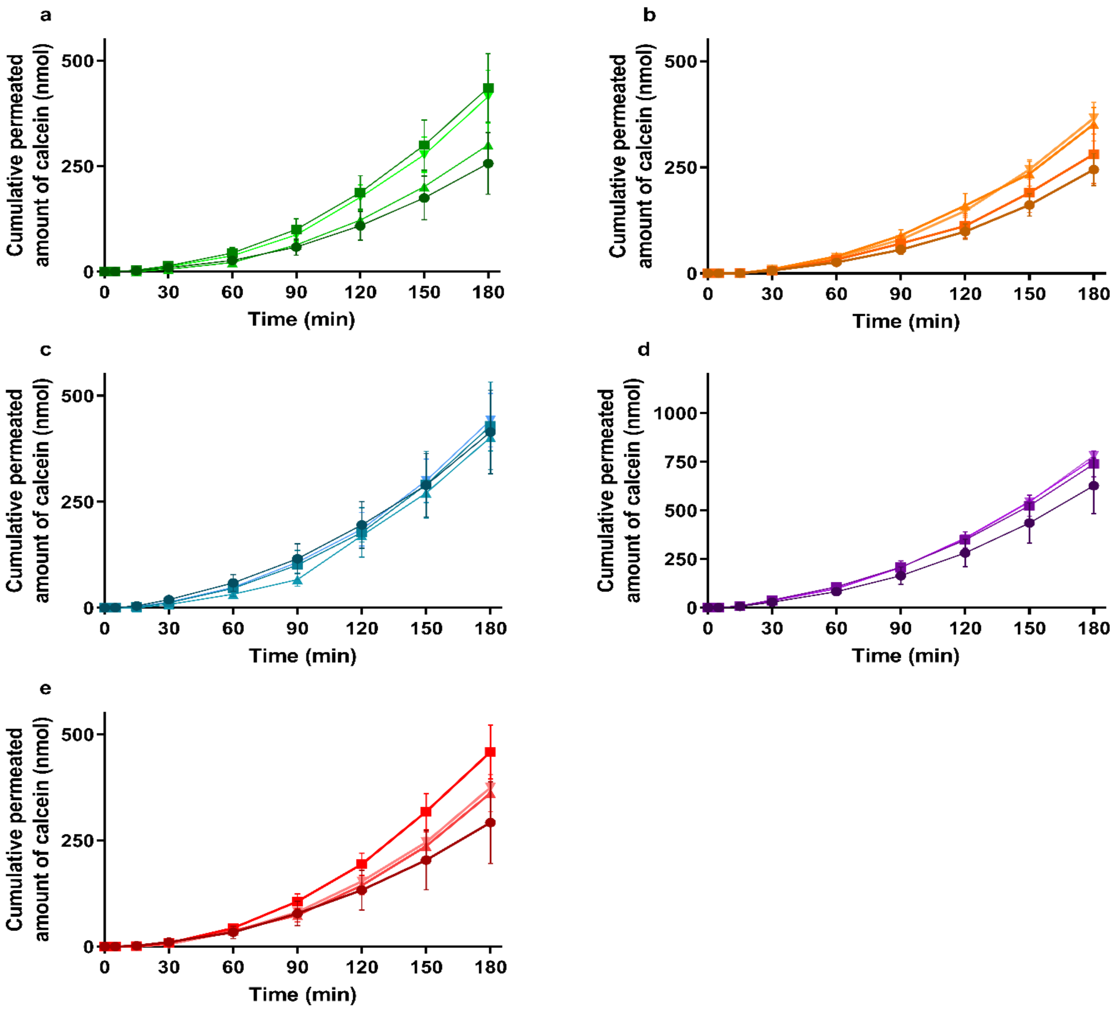

Figure S1. The individual permeation graphs with the cumulative permeated amount of calcein after testing the five formulations SNEDDS$_{80\%}$ (green) (**a**), Chasing principle (orange) (**b**), super-SNEDDS suspension (blue) (**c**), superSNEDDS solution (purple) (**d**), and aqueous suspension (red) (**e**) in the lipolysis-permeation method following 0 min (●), 15 min (■), 30 min (▲) and 60 min (▼) of lipolysis. The data is presented as mean ± SEM (n = 3).

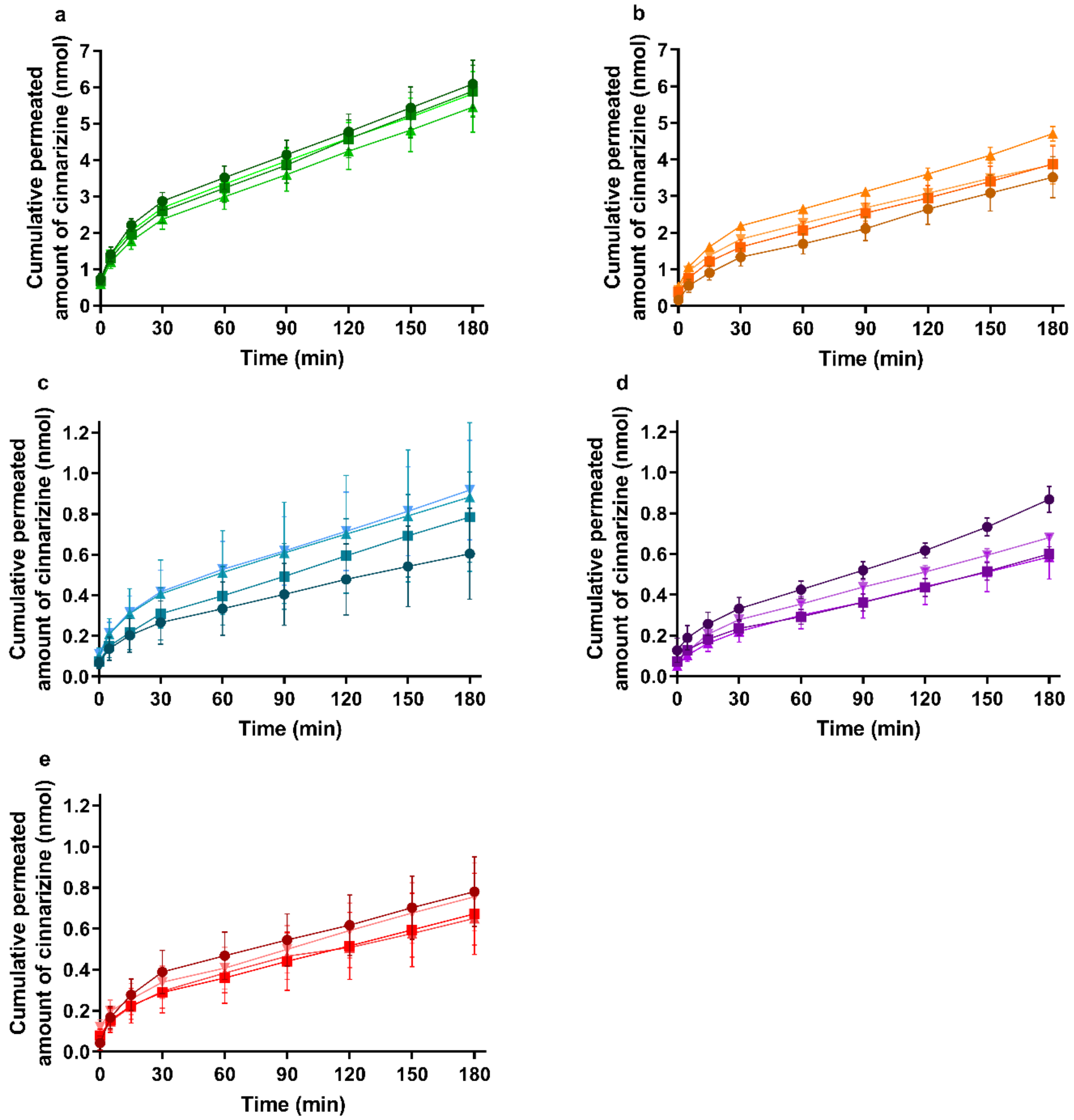

Figure S2. The individual permeation graphs with the cumulative permeated amount of cinnarizine after testing the five formulations SNEDDS$_{80\%}$ (green) (a), Chasing principle (orange) (b), superSNEDDS suspension (blue) (c), superSNEDDS solution (purple) (d), and aqueous suspension (red) (e) in the lipolysis-permeation method following 0 min (•), 15 min (■), 30 min (▲) and 60 min (▼) of lipolysis. The data is presented as mean±SEM (n = 3).

References

39. Siqueira, S.D.; Müllertz, A.; Gräeser, K.; Kasten, G.; Mu, H.; Rades, T. Influence of drug load and physical form of cinnarizine in new SNEDDS dosing regimens: In vivo and in vitro evaluations. *AAPS J.* **2017**, doi:10.1208/s12248-016-0038-4.
